# Polyphosphates as Inhibitors for Poly(vinyl Chloride) Photodegradation

**DOI:** 10.3390/molecules22111849

**Published:** 2017-10-28

**Authors:** Dina S. Ahmed, Gamal A. El-Hiti, Emad Yousif, Ayad S. Hameed

**Affiliations:** 1 Department of Chemistry, College of Science, Tikrit University, Tikrit 34001, Iraq; dinasaadi86@gmail.com (D.S.A.); ch@sc.nahrainuniv.edu.iq (A.S.H.); 2 Cornea Research Chair, Department of Optometry, College of Applied Medical Sciences, King Saud University, P.O. Box 10219, Riyadh 11433, Saudi Arabia; 3 Department of Chemistry, College of Science, Al-Nahrain University, Baghdad 64021, Iraq

**Keywords:** poly(vinyl chloride), functional groups indices, photodegradation, polyphosphates, inhibitors

## Abstract

Three polyphosphates were used as inhibitors for poly(vinyl chloride) (PVC) photodegradation. The polyphosphates were added to PVC at a concentration of 0.5% by weight. The PVC films (40 µm thickness) were irradiated at room temperature with ultraviolet (UV) light for up to 300 h. The changes in PVC films after irradiation were monitored by Fourier transform infrared spectroscopy, weight loss, viscosity-average molecular weight determination, and atomic force microscopy. These changes were very noticeable in the blank PVC films compared to the ones obtained when additives were used. The polyphosphates can inhibit the PVC photodegradation through direct absorption of UV light, interactions with PVC chains, and acting as radical scavengers.

## 1. Introduction

Recently, the principles of green chemistry have attracted noticeable attention [1,2]. Green chemistry tends to eliminate or reduce waste byproducts by developing chemical processes that are environmentally friendly [3]. Therefore, the production of chemicals and in particular polymers has to be efficient to produce materials that are highly stable and resist weathering conditions. Polymers and, in particular, poly(vinyl chloride) (PVC), are widely used in various applications. For example, PVC can be used in automobiles, pipes, electrical cables, window frames, packing, and many other applications. Linear PVC is a colorless rigid material with high density and a low softening point. PVC can be plasticized by the addition of plasticizers and is mainly used in construction applications. Natural or artificial ultraviolet (UV) light can make PVC photodegrade. This leads to physical and chemical changes within the polymeric chains [4,5]. Chlorine atoms increase the chain attraction and, therefore, enhance the hardness and stiffness of the polymer [6]. PVC undergoes dehydrochlorination, upon light exposure, to produce conjugated polyenes that lead to discoloration [7,8,9]. Therefore, the mechanical and physical properties of PVC have to be enhanced to resist the changes caused by irradiation or high temperature. Metal complexes and inorganic salts were used to stabilize PVC against irradiation, but the possible leakage of metals from such additives has never been tested for long-term use [10,11,12,13,14]. Moreover, a wide range of Schiff bases [15,16,17,18], aromatics [19,20,21], and heterocycles [22,23,24] have been used as inhibitors of PVC photodegradation.

Phosphorus organic polymers are highly stable and can be used for carbon dioxide gas storage or as surface adhesion agents, flame proofers, fire retardants, and catalysts due to their excellent physical and mechanical properties [25,26,27,28,29,30,31,32,33]. Also, they have been used in various biomedical applications [34,35]. Recently, we synthesized three new eco-friendly polyphosphates as efficient storage media for carbon dioxide [25]. It was of interest to test whether these polyphosphates could act as PVC photostabilizers against irradiation. We now report their successful use as new photostabilizers against PVC photodegradation upon irradiation with UV light, as a continuation of our research in the area of synthesis and the applications of polymers [36,37,38,39,40]. The efficiency of the polyphosphates used as photostabilizers for PVC was better than or comparable to that reported at the same concentration [10,15,18,20,21,22,23].

## 2. Results and Discussion

### 2.1. Synthesis of Polyphosphates ***1**−**3***

**Figure 1:**
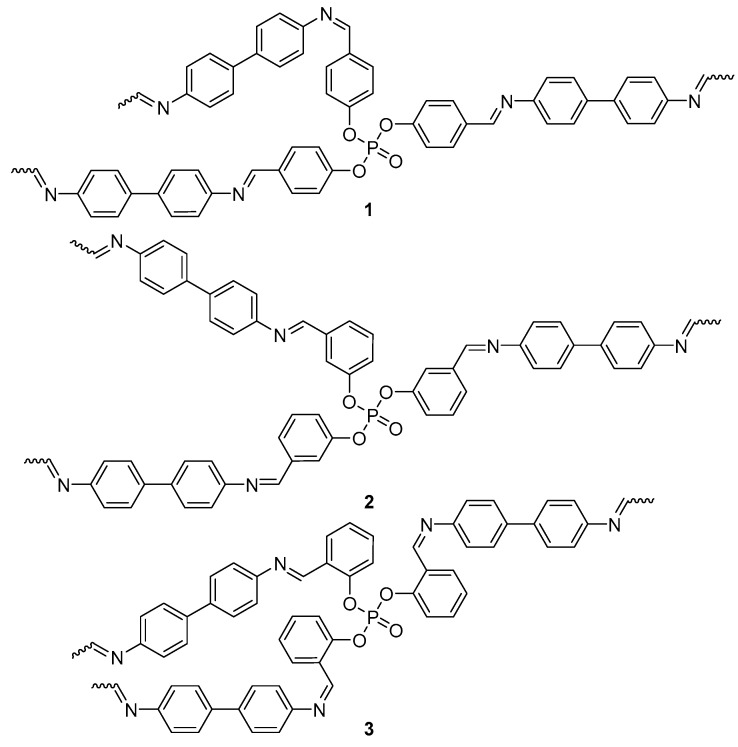
Polyphosphates **1**–**3**.

Polyphosphates **1**–**3** (Figure 1) were synthesized as previously reported [25] from reactions of *tris*(formylphenyl)phosphates, obtained from a reaction of appropriate hydroxybenzaldehyde (3 mole equivalents) and phosphoryl chloride with benzidine (1.5 mole equivalents) in chloroform in the presence of acetic acid under reflux for 6 h. The structures of **1**–**3** were confirmed by various spectral and analytical data and were consistent with those reported.

### 2.2. Determination of PVC Photodegradation by Fourier Transform Infrared (FTIR) Spectrophotometry

**Figure 2:**
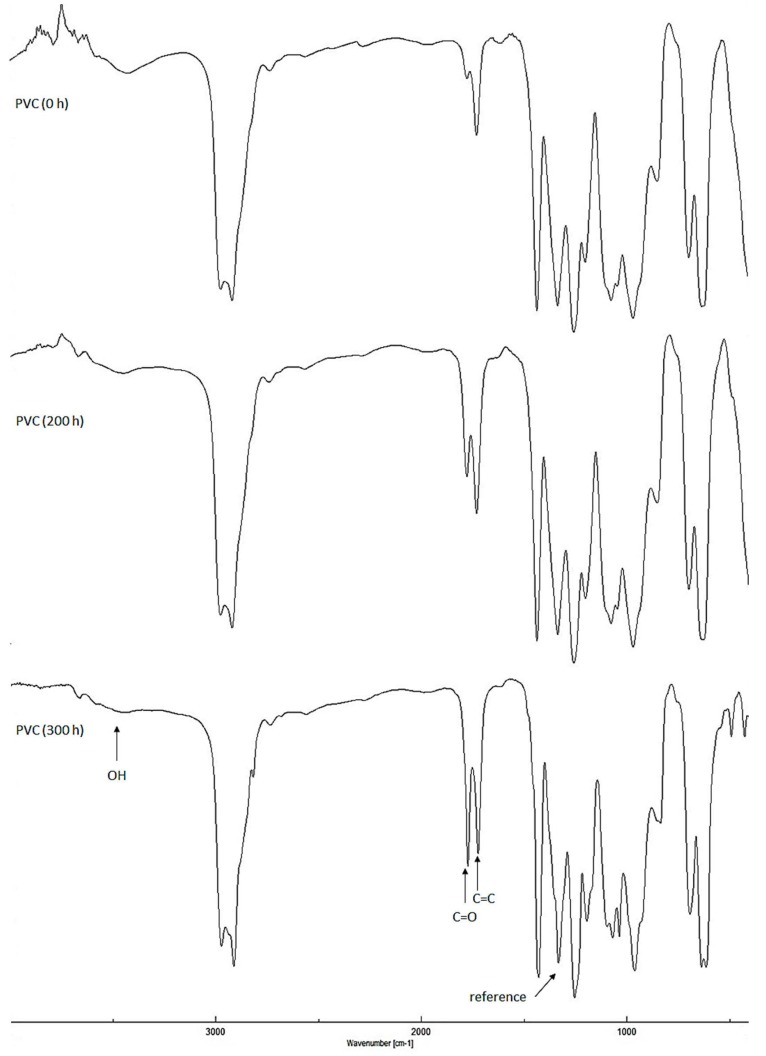
Effect of irradiation on infrared (IR) spectra of poly (vinyl chloride) (PVC) film.

The efficiency of polyphosphates **1**–**3** (0.5 wt %) as photostabilizers for PVC films (40 μm), upon irradiation with a UV light (λ_max_ = 365 nm), was investigated by FTIR spectroscopy. The infrared (IR) peak intensities for various functional groups (e.g., carbonyl, polyene and hydroxyl) for PVC films were different before and after irradiation (Figure 2).

**Figure 3:**
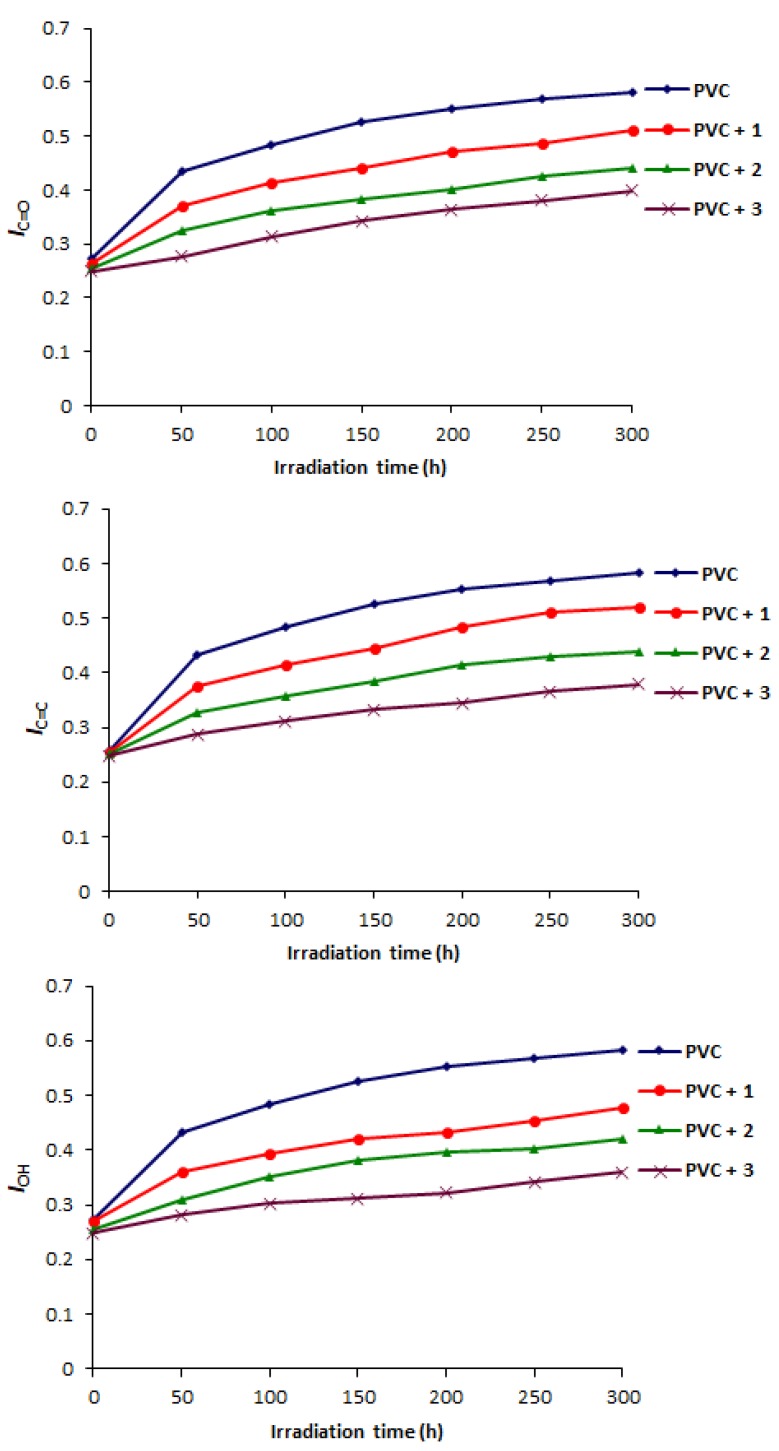
Effect of irradiation on *I*_C=O_, *I*_C=C_ and *I*_OH_ for PVC films.

Bands appearing at 1722, 1602, and 3500 cm^−1^ in the FTIR spectra of PVC films are attributed to aliphatic ketones, conjugated double bonds to a carbonyl group, and alcoholic hydroxy groups, respectively [16,41]. The PVC photodegradation was investigated by observing the variation in carbonyl, alkene and hydroxyl groups stretching absorption bands during irradiation process [42]. The changes in peak intensities for these functional groups were compared to a standard peak (1328 cm^−1^). The indices for the functional groups (*I*_C=O_, *I*_C=C_, and *I*_OH_) formation were calculated and plotted against irradiation time (Figure 3). The C=O, C=C, and OH indices for PVC films (40 μm) in the presence of polyphosphates **1**–**3** (0.5 wt %) were low compared to the ones for the blank PVC film (40 μm). Clearly, polyphosphates **1**–**3** inhibit photodegradation to a greater extent and can be as additives for long-term protection of PVC films against irradiation.

### 2.3. Determination of PVC Photodegradation by Weight Loss

**Figure 4:**
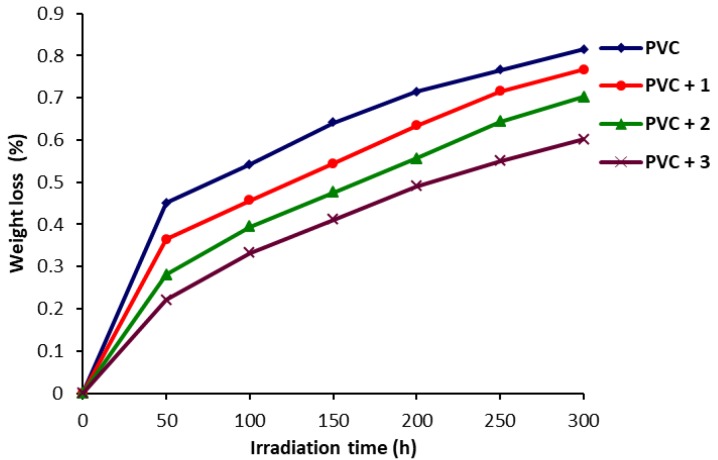
Effect of irradiation on weight loss (%) for PVC films.

Generally, organic UV-stabilizers are of low molecular weight and their addition to polymeric materials leads to various problems such as incompatibility, migration, and volatility [43]. Therefore, the use of high molecular weight UV stabilizers could be better for long-term protection of PVC against irradiation. The PVC films in the presence of polyphosphate **1**–**3** (0.5 wt %) were irradiated for 300 h and the loss in weight (%) was calculated and plotted against irradiation time (Figure 4). Obviously, the PVC weight loss percentage was lowest in the presence of **3** and highest for the blank PVC. The order of polyphosphates for the PVC photostabilization was **3** > **2** > **1**.

### 2.4. Determination of PVC Photodegradation by Viscometry Method

**Figure 5:**
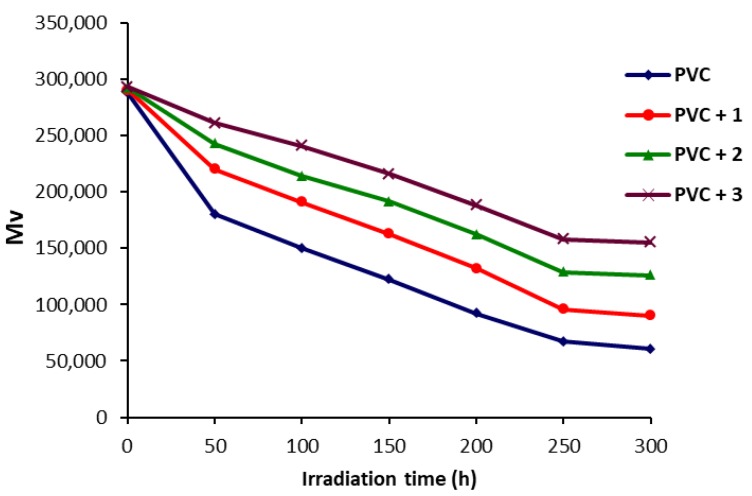
Effect of irradiation on viscosity-average molecular weight (${\bar{M}}_{V}$) for PVC films.

Covalent polymers have long or hyper-branched chains with high solubility and viscosity [44,45]. Polymers such as PVC can be characterized using viscometry method [46]. The viscosity-average molecular weight $\left({\bar{M}}_{V};\;g/\mathrm{mol}\right)$ of irradiated PVC films (40 μm) were determined from the intrinsic viscosity using the Mark–Houwink Equation [46,47]. Figure 5 shows the change in ${\bar{M}}_{V}$ with irradiation time for PVC films. The reduction in ${\bar{M}}_{V}$ was very low in the presence of polyphosphate **3** compared to the other PVC films.

Photodegradation of PVC under natural weathering or related laboratory conditions leads to crosslinking and chain scission [43]. Therefore, it is important to measure the average chain scission (*S*), which gives a clear picture of the degree of PVC photodegradation [48]. Equation (1) is used to calculate *S* from the viscosity average molecular weight at irradiation time *0* (${\bar{M}}_{V,O}$) and *t* (${\bar{M}}_{V,t}$) [49]:

(1) $$S=\;{\bar{M}}_{V,O\;}/{\bar{M}}_{V,t}\;-1.$$

**Figure 6:**
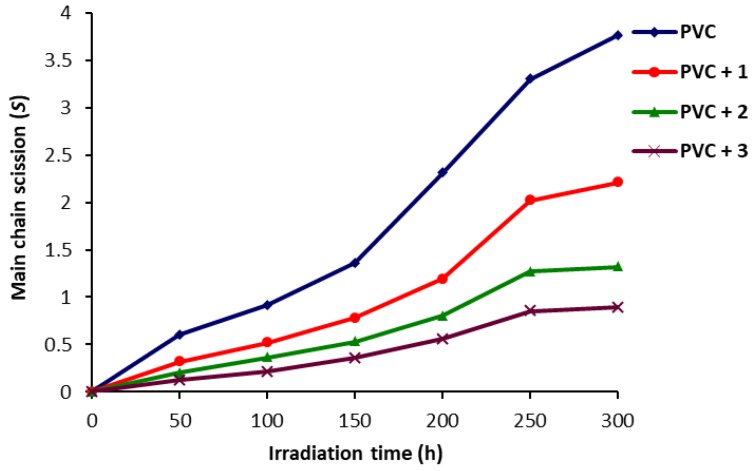
Effect of irradiation on *S* for PVC films.

The effect of irradiation time on *S* for PVC films is shown in Figure 6. Clearly, the main chain scission was very low for the PVC film containing polyphosphate **3**. Also, the quantity of insoluble materials produced during the photodegradation process was minimal when polyphosphate **3** was used as an additive and was significant in the case of the blank PVC. This is clear evidence that significant chain cross-linking has taken place in the case of the blank PVC. Also, it indicates that polyphosphates **1**–**3** can act as efficient inhibitors against photodegradation of PVC.

The degree of deterioration (*α*) gives an indication about randomly distributed weak bonds breaking which takes place at the initial stages of photodegradation. Equation (2) is used to calculate the values of *α* for PVC films during irradiation:

(2) $$\alpha =\left[{\bar{M}}_{V,0}\times S\right]/{\bar{M}}_{V,t}.$$

**Figure 7:**
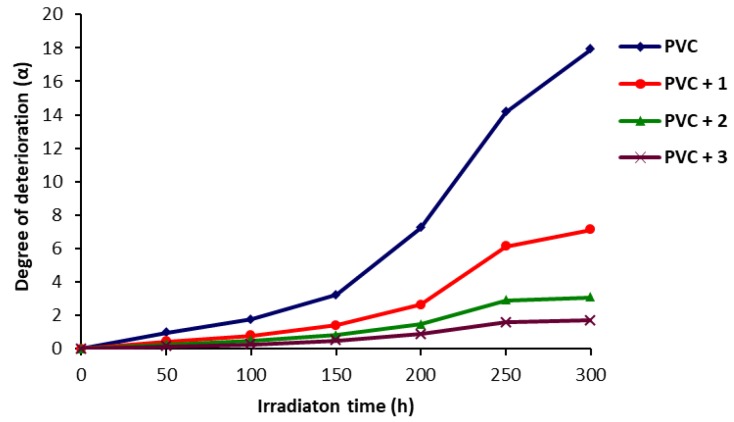
Effect of irradiation on *α* for PVC films.

The effect of irradiation time on *α* is shown in Figure 7. The curves indicate that the values of α for the irradiated PVC films (blank) were much higher than the ones obtained for PVC films in the presence of additives. The lowest values for *α* were seen when polyphosphate **3** was used as a photostabilizer. The random degradation of bonds was very slow in the first 150 h and then sharply increased with irradiation time.

### 2.5. Determination of PVC Photodegradation by Surface Morphology

**Figure 8:**
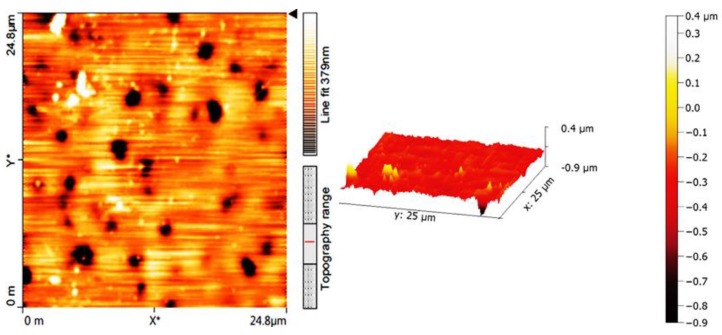
Atomic force microscopy (AFM) images of PVC film (control) after irradiation (300 h).

**Figure 9:**
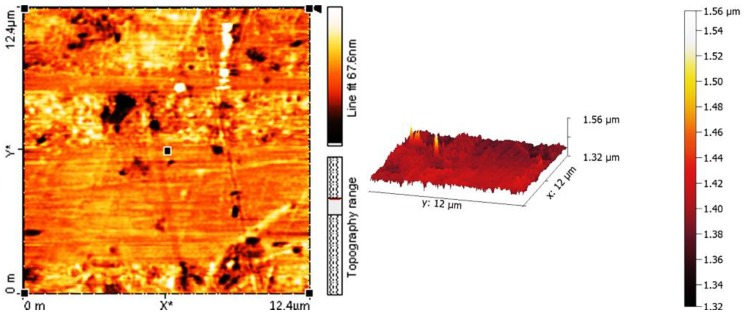
AFM images of PVC film containing **3** after irradiation (300 h).

Atomic force microscopy (AFM) is one of the most common techniques to examine the surface morphology of polymeric materials. The AFM images for the blank PVC film and the one containing polyphosphate **3**, after irradiation (300 h), are shown in Figure 8 and Figure 9, respectively. The black spots can be attributed to the polyphosphate chains and the residue from the PVC matrix. The surface of the PVC film containing **3** after irradiation was smooth; roughness (*R*_q_) was low (7.89 nm). In contrast, the blank PVC film after irradiation has a highly rough surface (*R*_q_ = 132.2).

**Figure 10:**
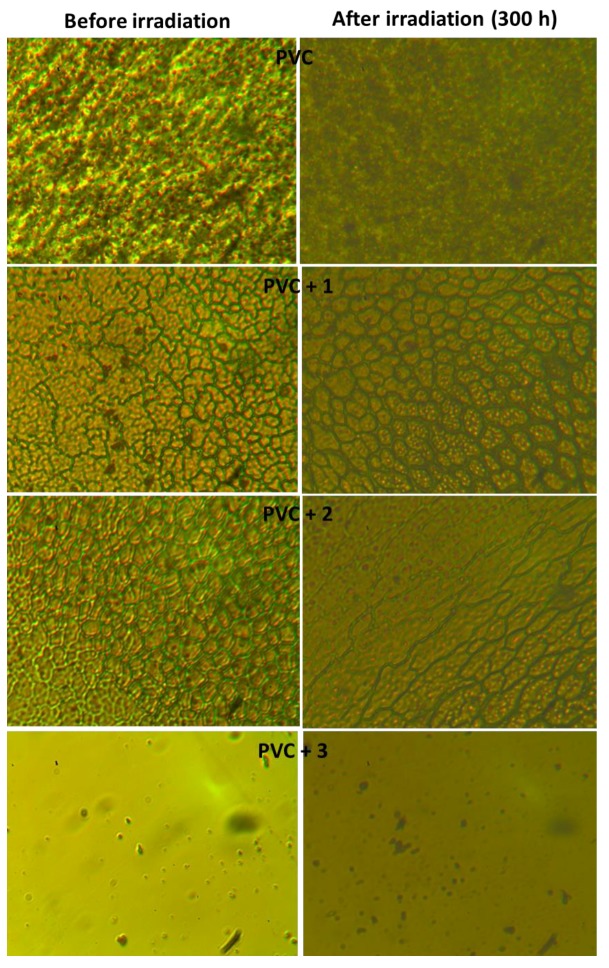
Effect of irradiation of PVC films on surface morphology.

UV irradiation of PVC leads to bond breakage and the removal of leachable constituents from the polymeric materials surface, causing a roughness in its surface [50]. In the absence of photostabilizers, photodegradation of PVC with a UV light leads to the elimination of hydrogen chloride (HCl), which is a common process at high temperatures [51]. It seems likely that polyphosphate **3** inhibits the dehydrochlorination process. The surface of PVC films before and after irradiation in the presence and the absence of polyphosphates **1**–**3** were examined using a microscope (Figure 10).

The surface of the irradiated (300 h) blank PVC film contains many white spots, holes, and grooves, which is a clear indication that photodegradation has taken place. In construct, the surface for the non-irradiated blank PVC was smooth, with no white spots or holes. In the case of PVC containing polyphosphates **1**–**3**, the surface was almost smooth with few white spots. Clearly, these additives act as efficient photostabilizers to protect the PVC films from deterioration upon irradiation. It should be noted that the differences among the PVC surfaces before irradiation (Figure 10) could be due to the manufacturing processes of the PVC films.

### 2.6. Suggested Mechanisms of PVC Photostabilization in the Presence of Polyphosphate

**Figure 11:**
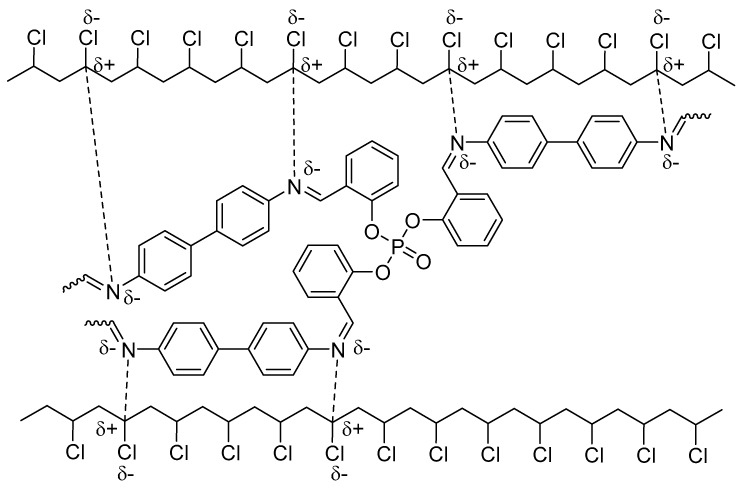
PVC photostabilization via interaction between PVC chains and polyphosphate **3**.

The synthesized polyphosphates dispersed uniformly in the PVC blends and acted as photostabilizers for PVC upon irradiation. The results show that the efficiency of such additives follows the order **3** > **2** > **1**. The CH=N bonds and aryl moieties in polyphosphates **1**–**3** play an important role in stabilizing the PVC against photodegradation. It is not clear why polyphosphate **3** was the most efficient additive. However, it is believed that the *ortho*-hydroxyl group attached to aromatic moieties within polyphosphate **3** makes such an additive an efficient radical scavenger compared to the other polyphosphates. Various mechanisms were suggested to explain the role that polyphosphates could have in the photostabilization of PVC films. The possible attraction between the polarized CH=N bonds within polyphosphates and the polarized C–Cl bonds within the PVC chains could stabilize PVC (Figure 11) through efficient transfer of the PVC excited energy to polyphosphates. Polyphosphates could then release such energy to heat overtime, which does not harm the PVC polymeric chains. However, the steric hindrance could reduce such a possibility.

**Figure 12:**
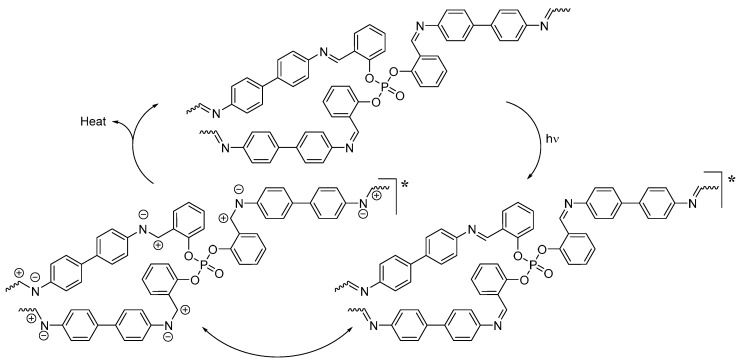
PVC photostabilization by polyphosphate **3** via direct absorption of UV radiation.

Aromatics commonly act as UV absorbers [19,20,21]. Polyphosphates **1**–**3** are electron-rich and can directly absorb the energy from UV light and dissipate it into heat energy at a level that is harmless to PVC (Figure 12).

**Figure 13:**
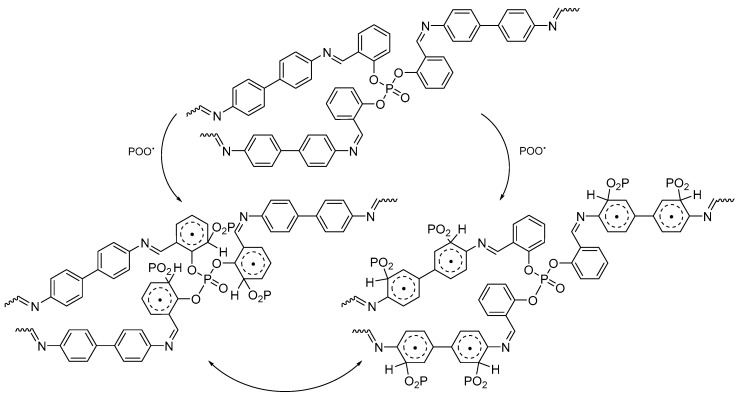
PVC photostabilization by polyphosphate **3** as a radical scavenger.

Polyphosphates **1**–**3** could act as radical scavengers to stabilize PVC (Figure 13) in the presence of a chromophore. Photodegradation of PVC leads to the formation of carbon radicals, which react with oxygen to produce peroxy radicals (POO^•^). Polyphosphates can form a stable complex with POO^•^ in which energy can be transferred. The stable charge transfer complex could be stabilized via the resonance of aryl rings in polyphosphates [52].

## 3. Experimental

### 3.1. General

PVC was purchased from Sigma-Aldrich Chemical Company (Gillingham, UK). Fourier transform infrared spectra (400–4000 cm^−1^) were recorded on a Jasco FT/IR-4200 spectrometer (Tokyo, Japan). An accelerated weather-meter QUV tester (Q-Panel Company, Homestead, FL, USA) was used to irradiate PVC films at room temperature with a UV light (λ_max_ = 365 nm and light intensity = 6.43 × 10^−9^ ein dm^−3^ s^−1^). The surface roughness of PVC films was detected by atomic force microscopy using a Veeco instrument (Plainview, NY, USA). The morphology images of PVC films were obtained using a Meiji Techno Microscope (Tokyo, Japan). A Digital Vernier Caliper 2610A micrometer (Vogel GmbH, Kevelaer, Germany) was used to prepare PVC films (40 μm) containing polyphosphates (0.5 wt %). The films were produced using the evaporation technique (room temperature, 24 h). The PVC films were fixed using aluminum plate stands with a thickness of 0.6 mm (Q-Panel Company, Homestead, FL, USA).

### 3.2. Preparation of PVC Films

A solution of PVC (5 g) in tetrahydrofuran (100 mL) was stirred for 30 min at room temperature. Polyphosphates **1**–**3** (0.5 wt %) were added to the PVC solution and the mixture was stirred for 30 min at room temperature. The mixture was cast onto glass plates to allow the solvent to evaporate at room temperature for 24 h. Polyphosphates **1**–**3** were found to be completely miscible with PVC.

### 3.3. Determination of PVC Photodegradation by FTIR Spectrophotometry

Ultraviolet radiation of polymeric materials leads to changes in their chemical, mechanical, and physical properties [53]. Photo-oxidation of PVC leads to the formation of carbonyl, conjugated double bonds, and hydroxyl moieties [16,54,55,56]. Therefore, the changes in intensity of carbonyl (1722 cm^−1^), polyene (1602 cm^−1^), and hydroxyl (3500 cm^−1^) peaks in the IR spectra for PVC after irradiation were used to measure the degree of photodegradation. A reference peak that appears at 1328 cm^−1^, due to the C–C bond within PVC chains, was used for comparison [57]. Such a peak was not affected by UV irradiation. The functional group index (*I*_S_) for each functional group was calculated using Equation (3), where *A_s_* is the peak absorbance for a specific functional group and *A_r_* is the absorbance for the reference peak:

(3) $$I_{s}=A_{s}/A_{r}.$$

### 3.4. Determination of PVC Photodegradation by Weight Loss

The weight loss (%) was calculated from the PVC weights before irradiation (*W*_1_) and after irradiation (*W*_2_) using Equation (4) [24]:

(4) $$Weight\;loss\;\%\;=\;\left[\left(W_{1}-W_{2}\right)/W_{1}\right]\;\times \;100.$$

### 3.5. Determination of PVC Photodegradation by the Viscometry Method

The average molecular weight (${\bar{M}}_{V}^{\alpha }$) of PVC films is calculated from the intrinsic viscosity, [*η*], using Equation (5), where both *α* and *K* are constants [58]:

(5) $$\left[\eta \right]=K{\bar{M}}_{V}^{\alpha }.$$

## 4. Conclusions

Three polyphosphates were used as photostabilizers of PVC films. FT-IR spectroscopy and viscosity-average molecular weight analysis indicated that polyphosphates act as inhibitors of PVC photodegradation. The atomic force microscopy and microscope analyses showed that the surface of the PVC films containing polyphosphates were smoother and contained fewer cracks compared to the PVC (blank). Polyphosphates could be used as commercial additives for longer term protection of PVC against UV irradiation. Various mechanisms were suggested to explain the role polyphosphates could have in the stabilization PVC. However, detailed mechanisms based on quantum chemistry calculation are still needed.
